# Supplementary material for: Generalizing the isothermal efficiency by using Gaussian distributions
Source: PLoS One. 2023 Jan 10;18(1):e0279758. doi: 10.1371/journal.pone.0279758 (PMC9831307; doi:10.1371/journal.pone.0279758)
Supplement: Attachment — Submitted filename: ReviewPLOSONE.docx [file pone.0279758.s002.docx]

Referee Report regarding the manuscript entitled “**Generalizing the isothermal efficiency by using Gaussian distributions**”

In this manuscript, the author has shown that the channel capacity and the isothermal efficiency equations can be derived without using thermodynamics from the assumption of Gaussian noise impinging on a high dimensional system. The manuscript is well written and the presented derivation is novel. The importance of this study is that since Gaussian noise and high dimensionality are found at all levels of biology, the channel capacity and the isothermal efficiency equations can be applied universally to understand biological states. Consequently, I recommend this manuscript for a possible publication in the journal PLOS ONE.
